# A hydrophobic barrier deep within the inner pore of the TWIK-1 K2P potassium channel

**DOI:** 10.1038/ncomms5377

**Published:** 2014-07-08

**Authors:** Prafulla Aryal, Firdaus Abd-Wahab, Giovanna Bucci, Mark S. P. Sansom, Stephen J. Tucker

**Affiliations:** 1 Clarendon Laboratory, Department of Physics, University of Oxford, Oxford OX1 3PU, UK; 2 Department of Biochemistry, University of Oxford, Oxford OX1 3QX, UK; 3 OXION Initiative in Ion Channels and Disease, University of Oxford, Oxford OX1 3PT, UK

## Abstract

Recent X-ray crystal structures of the two-pore domain (K2P) family of potassium channels have revealed a unique structural architecture at the point where the cytoplasmic bundle-crossing gate is found in most other tetrameric K^+^ channels. However, despite the apparently open nature of the inner pore in the TWIK-1 (K2P1/KCNK1) crystal structure, the reasons underlying its low levels of functional activity remain unclear. In this study, we use a combination of molecular dynamics simulations and functional validation to demonstrate that TWIK-1 possesses a hydrophobic barrier deep within the inner pore, and that stochastic dewetting of this hydrophobic constriction acts as a major barrier to ion conduction. These results not only provide an important insight into the mechanisms which control TWIK-1 channel activity, but also have important implications for our understanding of how ion permeation may be controlled in similar ion channels and pores.

## Abstract (web summary)

K2P potassium channels have a structure dissimilar to other potassium channels. Here, the authors study the K2P channel TWIK-1 and show that the protein contains a deep pore hydrophobic barrier that blocks ion channel conductance.

## Graphical abstract

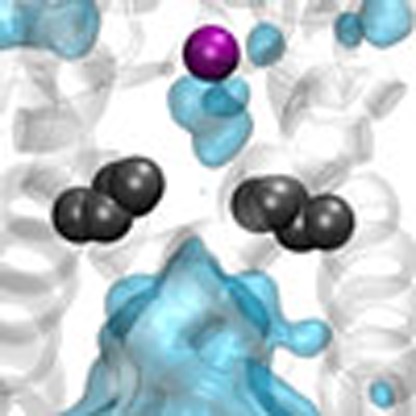

K2P potassium channels represent a structurally unique family of channels involved in physiological functions as diverse as cell-volume regulation, central chemosensitivity, apoptosis, vasodilatation, neuronal excitability and the perception of pain^1^. They also act as a major target of volatile anaesthetics and represent attractive therapeutic targets for the treatment of a variety of cardiovascular and neurological disorders^2^,^3^. In particular, the first mammalian member of this superfamily to be cloned, TWIK-1 (K2P1/*KCNK1*), has been implicated in having an important physiological role in the brain and the heart^4^,^5^,^6^. However, understanding the biophysical, pharmacological and functional properties of this particular channel has been difficult. This is mainly because of the extremely low levels of functional activity observed when TWIK-1 is expressed in heterologous systems, especially when compared with other members of the K2P family^7^. The precise reasons for this remain controversial and may involve a combination of either post-translational modification (sumoylation)^8^,^9^ and/or rapid endocytosis^10^,^11^,^12^,^13^. However, it is clear that even when TWIK-1 channels are present in the plasma membrane they have very low levels of intrinsic functional activity and are effectively silent^10^,^11^,^12^,^14^.

Two recent crystal structures of K2P channels (TWIK-1 and TRAAK) revealed that these channels assemble as a dimer with many similarities to classical K^+^ channels including a ‘pseudotetrameric’ K^+^ selective pore^15^,^16^. However, functional studies have indicated that K2P channels, unlike most other K^+^ channels, do not utilize an intersection of the pore-lining helices or cytoplasmic ‘bundle-crossing’ as their primary gate. Instead, K2P channel gating is primarily the result of a gate located close to, or within, the selectivity filter^17^,^18^,^19^. It was therefore interesting to observe in the crystal structures of both TWIK-1 and TRAAK that the dimensions of the cytoplasmic entrance to the pore were comparable to other K^+^ channel structures believed to represent functionally open conformations^20^,^21^. However, despite the apparently ‘open’ conformation of the TWIK-1 structure, the arrangement of the transmembrane helices at the cytoplasmic entrance to the pore remains unusual; the distal end of M4 is kinked parallel to the membrane forming an amphipathic ‘C-helix’ that has been proposed to have a role in channel gating^16^. In addition, the C-helix also contains Lys274, the residue proposed to be the site responsible for channel ‘silencing’ by post-translational sumoylation^8^,^9^.

In this study we sought to investigate whether the structural features of TWIK-1 may provide an insight into the apparent lack of functional activity when this channel is expressed. We therefore employed multi-scale molecular dynamics (MD) tools to simulate the behaviour of the TWIK-1 crystal structure within a lipid membrane environment. Intriguingly, we found evidence for a hydrophobic gate deep within the inner cavity of the TWIK-1 pore, and we demonstrate that this hydrophobic barrier plays a key role in determining the functional activity of TWIK-1.

## Results

### MD simulation of TWIK-1 within a lipid bilayer

**Figure 1:**
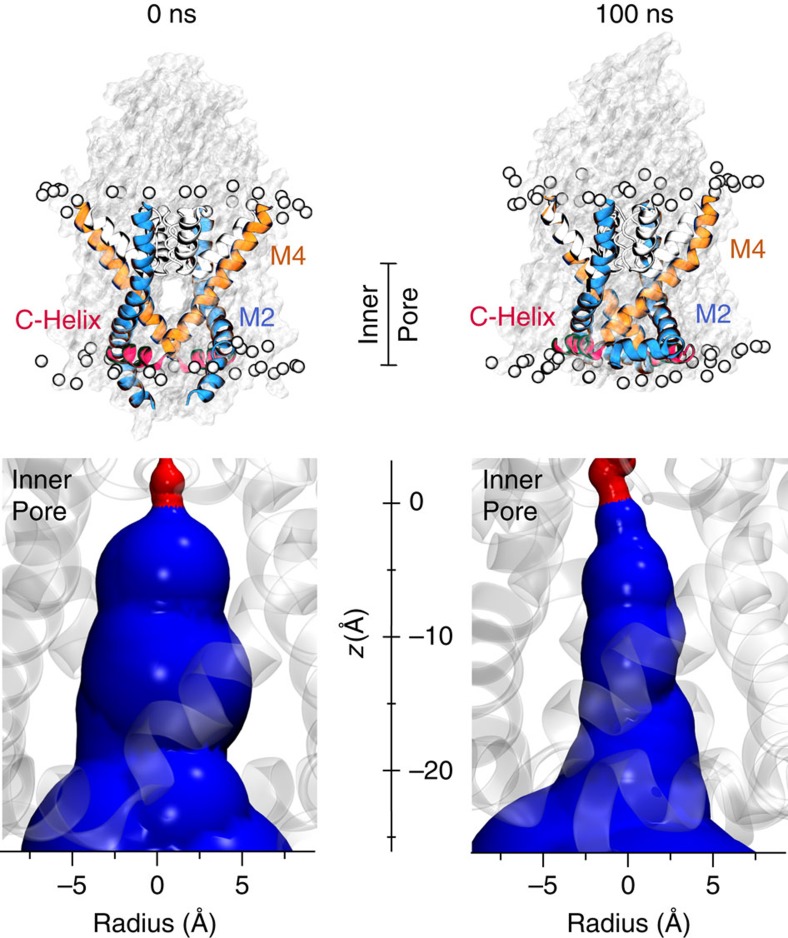
TWIK-1 cytoplasmic gate remains open. Top: pore-lining structure and surface of TWIK-1 at the start (0 ns), and end (100 ns) of the MD simulation. The pore-lining helices and C-helix are highlighted. The position of the membrane is shown by the phosphorus atoms of POPC highlighted as light grey spheres. The C-helix remains stable and associated with the membrane throughout the simulation (Supplementary Fig. 1). Below each snapshot of the simulation is an expanded view of the inner-pore section indicated by the line in the top panel. Distances along the *z*-axis of the inner pore start below the S4 binding site. The pore radius dimensions show that the cytoplasmic mouth of the inner cavity remains open throughout the simulation. The HOLE surface profiles are coloured red (*r*<1.2 Å) or blue (*r*>1.2 Å).

To simulate the behaviour of TWIK-1 within a lipid membrane environment we first used coarse-grain MD simulations (CGMD) to embed and equilibrate the experimentally determined channel structure in a phospholipid (POPC (1-palmitoyl,2-oleoyl-sn-glycero-3-phosphocholine)) bilayer. In the CGMD simulations and at the start of the subsequent atomistic simulations, the C-helix aligned parallel to the membrane and the side-fenestration between M2 and M4 was still visible (Fig. 1). At the end of the 100-ns atomistic simulation, movement of the transmembrane helices at the lipid interface resulted in a closure of the fenestration and a small upward movement of the distal end of M2. Nevertheless, the overall structure remained stable; in particular, the C-helix remained parallel to the plane of the membrane and the cytoplasmic gate and inner pore remained apparently open (Fig. 1; Supplementary Fig. 1).

### Water dynamics within the inner pore

**Figure 2:**
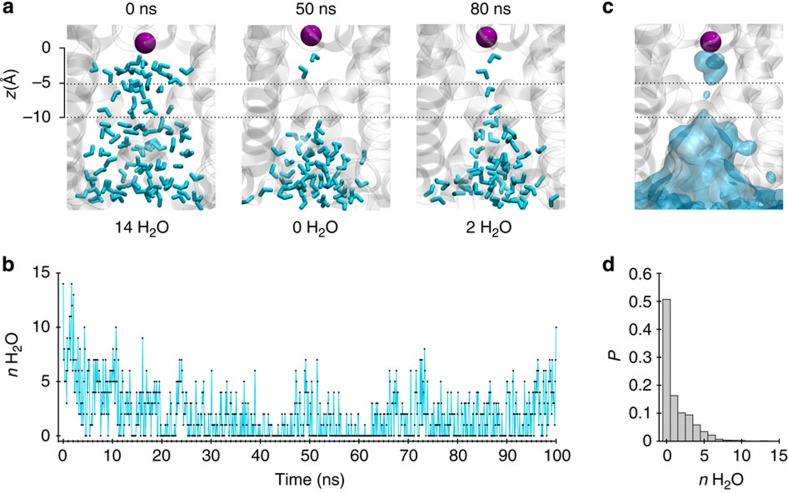
Dewetting of the inner pore. (**a**) Snapshots of water molecules (cyan) inside the inner pore of TWIK-1 channel at different time points during the MD simulation. For orientation, the K^+^ ion at the S4 site is shown as a purple sphere. Dashed lines (5–10 Å below the S4 site) indicate the location where dewetting is most prominent. The number of water molecules in this region is indicated below each snapshot. (**b**) Water count within this region of the inner pore sampled every 0.1 ns during the simulation. (**c**) Average water density inside the inner pore during the simulation is shown as transparent cyan surface contoured at 0.50 of bulk water density, overlaid on a snapshot of the inner pore at 100 ns. (**d**) Normalized probability histogram of the water count at this region for two 100-ns MD simulations. This reveals water molecules to be absent from this region of the inner pore for >50% of the duration of the simulation.

Closer examination of the simulation revealed an unusual behaviour of water molecules within the pore of the channel; a stochastic wetting and dewetting process was observed within the inner pore (Fig. 2; Supplementary Movie 1). Visualization of the averaged water density within the inner pore revealed that the water loss was most drastic around 5–10 Å below the selectivity filter (Fig. 2a). The number of water molecules within this region dropped to zero within a few nanoseconds of starting the simulation, followed by fluctuations throughout the simulation (Fig. 2a,b). An averaged distribution plot shows a clear absence of water within this region compared with the cytoplasmic entrance of the pore (Fig. 2c). Similar water dynamics were also observed in another separate 100-ns simulation and a fractional occupancy histogram shows that water was completely absent from this region of the inner pore for >50% of the duration of the simulations (Fig. 2d).

### Hydrophobic cuff deep within the TWIK-1 pore

**Figure 3:**
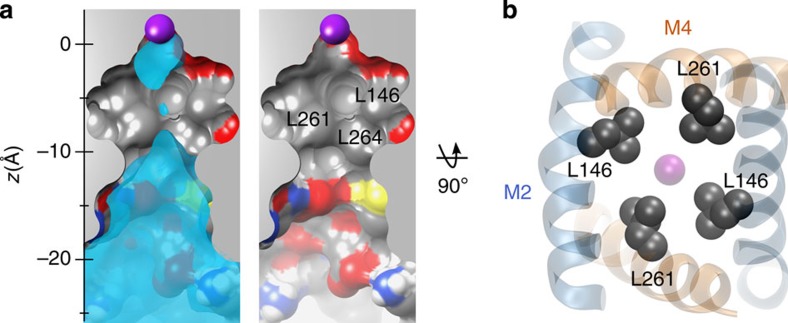
Hydrophobic cuff within the TWIK-1 inner pore. (**a**) A longitudinal section through the centre of the TWIK-1 channel at the end of the MD simulation. Left, a section of the average water density is overlaid. The S4 K^+^ ion is shown as a purple sphere. Note the hydrophobic nature residues lining the inner pore. Right, leucine residues contributing to the hydrophobic cuff are labelled. (**b**) Bottom-up view of the inner pore. Leu146 and Leu261 which form the hydrophobic cuff are shown as grey van der Waals spheres. M2 is shown in blue and M4 in orange.

To understand this dewetting process we examined the structure of the TWIK-1 pore. A section through the inner pore shows that the region where dewetting is most prominent is extremely hydrophobic (Fig. 3a). In particular, Leu146 and Leu261 from both subunits form a hydrophobic cuff around this region with their side chains pointing into the pore throughout the simulation (Fig. 3b). Another hydrophobic side chain (Leu264) also appears to contribute to this cuff, but this residue is located at the interface between M2 and M4, and so the side chain is not fully exposed to the conduction pathway. Interestingly, the hydrophobic nature of this inner cavity is in marked contrast to the cytoplasmic mouth of the pore which is lined by numerous polar side chains and which remains hydrated throughout the simulations (Fig. 3a).

### Simulations of inner pore hydration

Confinement of water and ions at the molecular scale can strongly influence their behaviour and lead to effects not anticipated from macroscopic descriptions^22^,^23^,^24^,^25^,^26^. The dewetting process we observed within the hydrophobic cuff therefore led us to consider the influence of a possible ‘hydrophobic gating’ process within the TWIK-1 pore, and to ask whether this hydrophobic barrier might contribute to the very low basal levels of ionic currents which these channels appear to conduct. The principle of hydrophobic gating was first demonstrated in MD simulations of model nanopores with hydrophobic constrictions of radius<6 Å where water begins to evaporate and fluctuate between its liquid and vapour states. These fluctuations lead to an effective energy barrier for ion permeation even without physical occlusion of the pore and therefore can function as a ‘vapour-lock’ gate^23^,^24^,^25^,^26^,^27^. This type of gating has now been shown to occur in several classes of ion channel including pentameric ligand-gated channels, the prokaryotic mechanosensitive MscS channel and voltage-gated K^+^ channels^28^,^29^,^30^,^31^.

**Figure 4:**
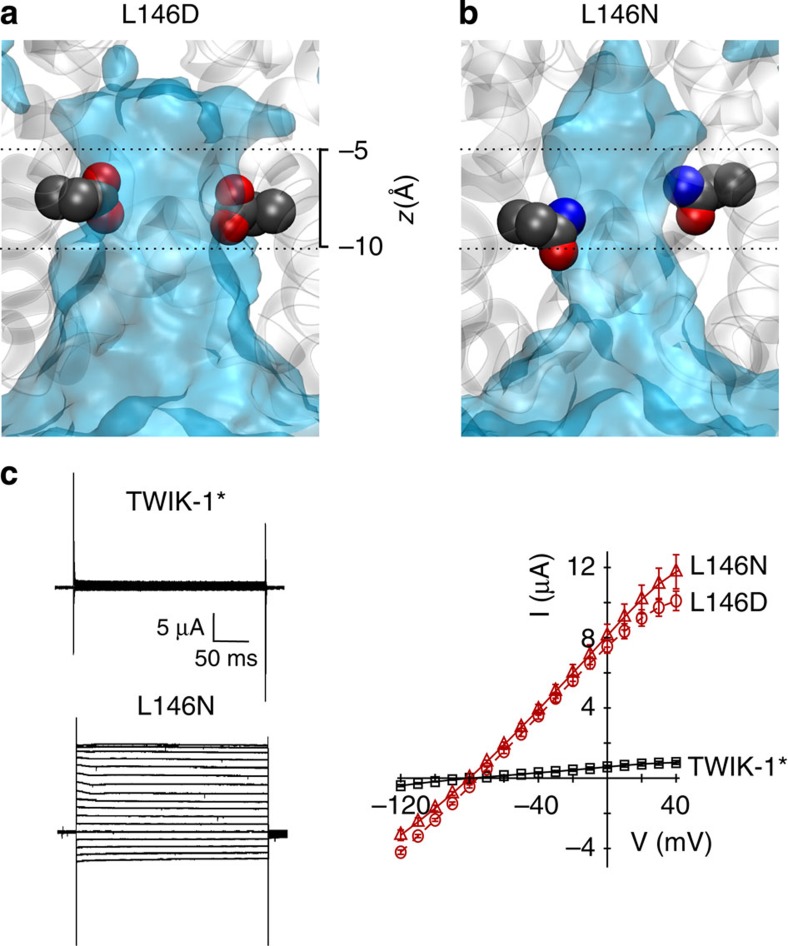
Mutation of Leu146 hydrates the inner pore. Average water density from a 100-ns MD simulation of: (**a**) L146D and (**b**) L146N mutants. The heavy atoms of the mutant side chains are shown as van der Waals spheres at the end of the MD simulation (see also Fig. 2c). Dashed lines represent approximate position of the hydrophobic cuff (**c**) Left, representative whole-cell currents recorded from the WT TWIK-1* and L146N-mutant channels. Right, averaged current–voltage relationship for whole-cell currents of WT TWIK-1* (black), L146D and L146N TWIK-1* mutants (red) expressed in *Xenopus* oocytes.

Intriguingly, a previous study showed that the L146D mutation in M2 of TWIK-1 resulted in large currents when expressed in *Xenopus* oocytes^11^. This result is consistent with Leu146 contributing to a hydrophobic barrier within the pore and with disruption of this barrier by the L146D mutation. We therefore examined the water dynamics of a TWIK-1 channel with the L146D mutation during a 100-ns simulation. This mutation markedly altered the behaviour of water within the inner pore, which remained hydrated throughout the simulation (Fig. 4a; Supplementary Fig. 2). However, the L146D mutation introduces a negative charge into the pore. We therefore changed Leu146 to another hydrophilic, but non-charged side chain of similar size (L146N), and repeated the MD simulation. Analysis of water occupancy and density revealed that the L146N mutant also retained water within the inner pore, indicating that this effect is not just the result of introducing a negative charge into the pore (Fig. 4b; Supplementary Fig. 2).

**Figure 5:**
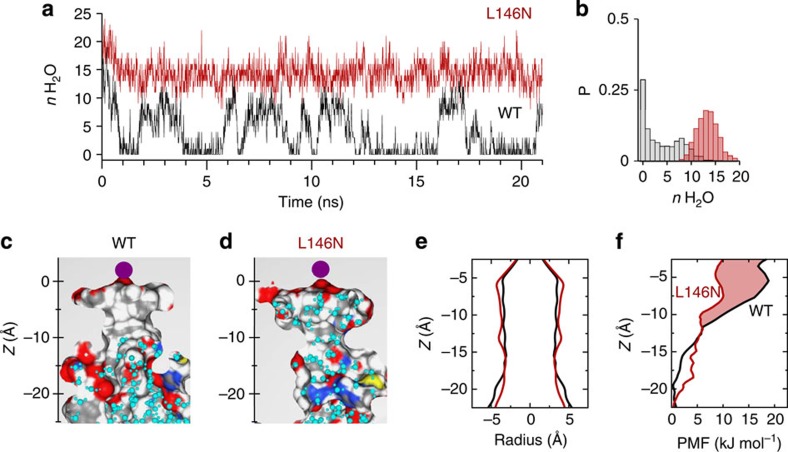
Positional restraint simulations and energetic profiles. Dewetting precedes structural changes in the inner pore during the soft positional restraint simulation (**a**) Water count within the hydrophobic gate region (as in Fig. 2b) during a positional restraint simulation for WT (black) and L146N (red) sampled every 0.01 ns. Positional restraint with a force constant of 10 KJ mol^−1^ Å^−2^ were applied to the Cα carbons of WT and *in-silico* L146N mutants (**b**) Normalized probability histogram of the water count at the hydrophobic cuff during the positional restraint simulations. This shows that ~30% of the simulation time, there is no water in the hydrophobic region (Δ*μ* *n*H_2_O _(L146N–WT)_=10.37). (**c**) and (**d**) Cross-section of inner pore of the starting structures for probability mass function calculations where *n*H_2_O_(WT)_=0 and *n*H_2_O_(L146N)_=12. (**e**) The starting PMF structures, WT structure (black) and L146N structure (red) have a comparable radius profile. (**f**) Potential mean force profile for a K^+^ ion translated along the *z*-axis of the inner pore of WT channel (black) and L146N-mutant channel (red). The shaded red area represents the additional free energy barrier experienced in the WT channel relative to the L146N-mutant within and above the hydrophobic cuff.

As a further control, we also performed MD simulations of the wild-type (WT) and L146N-mutant TWIK-1 channels using a variety of different force fields. Importantly, dewetting was observed in the WT channel irrespective of the force field used, whereas the L146N-mutant simulations all remained fully hydrated (Supplementary Fig. 3). Furthermore, to determine whether this dewetting process was a result of subtle changes in pore structure during the simulations, we performed additional simulations in which the Cα backbone atoms of the protein were subjected to a soft positional restraint, thus keeping the channel conformation close to that in the crystal structure. For the WT structure, a similar dewetting process was also observed within the first few nanoseconds, followed by intermittent fluctuations of water within the inner cavity, whereas the L146N-mutant pore remained hydrated throughout (Fig. 5a–d). This demonstrates that dewetting of the WT channel is not a consequence of any of the minor structural changes which occur during the simulation. Furthermore, similar to the result seen during simulation of the NavMs sodium channel^32^, even though the fenestrations remained open during this restrained simulation of TWIK-1, the alkyl chains of the surrounding lipid bilayer were not long enough to occlude the inner cavity.

### Energetics of the hydrophobic barrier

We next reasoned that retention of water within the inner pore by the L146N mutation should also reduce the energetic barrier to the movement of a K^+^ ion through the inner cavity. We therefore performed potential mean force (PMF) calculations, which provide an estimate of the free energy profile for the transit of a K^+^ ion through the inner pore in equilibrated MD structures for WT and L146N-mutant structures (Fig. 5e). These results show that for the WT TWIK-1 structure there is a steadily increasing barrier to permeation as the ion approaches the hydrophobic cuff from the cytoplasmic entrance (Fig. 5f). This barrier continues towards the selectivity filter due to the associated dewetting of the region above the hydrophobic cuff. However, for the L146N mutation there is an overall reduction in this barrier (by about 4 kT), especially within the deep inner pore in the vicinity of this mutation. This demonstrates that the L146N mutation can directly influence the conduction pathway for K^+^ ions.

### L146N mutation increases channel activity

The result of these simulations therefore led us to test the effects of the hydrophilic L146N mutation on the functional activity of TWIK-1. To achieve this we used a variant of TWIK-1, which contains a trafficking mutation (I293A/I294A) within the cytoplasmic domain. This trafficking mutation (hereafter referred to as TWIK-1*) has been shown to prevent the endocytic retrieval of TWIK-1, thereby promoting stable expression of TWIK-1 channels within the plasma membrane^11^,^12^. As shown previously, we found that WT TWIK-1 produces no detectable channel activity when expressed in *Xenopus* oocytes and that the activity of the TWIK-1* mutant is barely detectable above background (Supplementary Fig. 4). This supports previous studies which show that even when TWIK-1 channels can reach the membrane they generally exhibit very low levels of channel activity^10^,^11^,^12^.

However, when the L146N mutation was introduced into TWIK-1*, we observed high levels of whole-cell current, similar to reported effects of the L146D mutation (Fig. 4c). This demonstrates that introduction of an uncharged hydrophilic side chain into the hydrophobic inner pore of TWIK channel not only leads to an increase in hydration of the inner cavity *in silico* but also to enhanced whole-cell currents. This result is also consistent with the effects of introducing similar polar side chains into the hydrophobic gate of the MscS channels^29^.

### Direct effects on the ion conduction pathway

Previous studies have shown that mutation of Leu146 and other residues within M2 do not affect the protein expression levels of TWIK-1* in *Xenopus* oocytes^11^. However, to confirm that the enhancement of whole-cell currents seen with the L146N-mutant channel is primarily due to a direct effect on the structure of the inner pore, we recorded currents in giant excised patches. We have previously used this approach to demonstrate that some K2P channels are sensitive to intracellular block by large quaternary ammonium ions (for example, tetrahexylammonium (THexA)), which bind deep within the inner cavity just below the selectivity filter. This property can then be used as a direct measurement of the structure of the inner cavity which forms part of the conduction pathway^18^,^19^.

**Figure 6:**
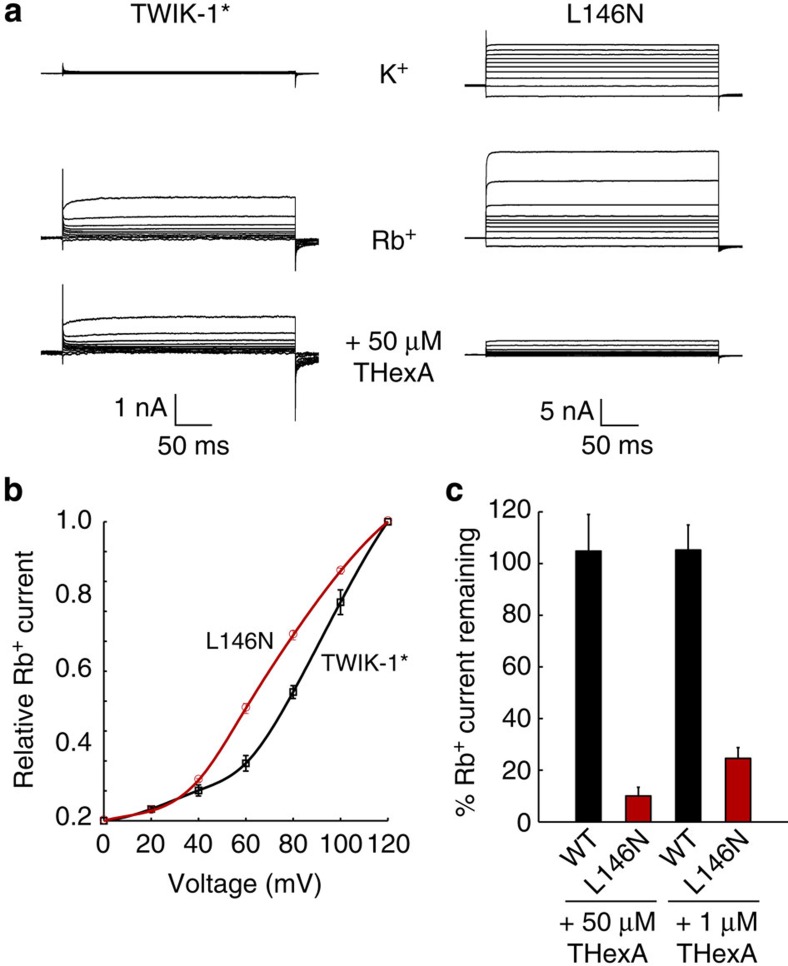
L146N mutation directly alters conduction pathway. (**a**) Typical currents recorded from giant excised patches expressing WT TWIK-1*. Almost no current can be recorded with K^+^ as the permeant ion in the intracellular bath solution. But replacing K^+^ in the bath by Rb^+^ produced large currents at depolarizing voltages. These TWIK-1* Rb^+^ currents are sensitive to block by quinine (not shown), but are not inhibited by 50-μM THexA. Similar recordings of L146N-mutant channels exhibit larger currents with both K^+^ and Rb^+^ as the permeant ions, but the L146N Rb^+^ currents are almost completely inhibited by 50-μM THexA applied intracellularly. (**b**) Voltage dependence of Rb^+^ currents for both wild-type TWIK-1* and L146N normalized to activation at +120 mV. Note that L146N currents activate over a lower voltage range. (**c**) Percentage remaining current for WT TWIK-1* and L146N-mutant channels after block by 50-μM and 1-μM THexA; L146N channels exhibit a markedly enhanced sensitivity to THexA.

We therefore first measured WT TWIK-1* currents in giant excised patches and found the channels to be essentially silent in symmetrical K^+^ solutions (Fig. 6a). However, TWIK-1 currents have been shown to be enhanced in the presence of Rb^+^ ions^14^ and so we measured TWIK-1* currents using Rb^+^ as a permeant ion. Under these conditions we were able to record outward currents through WT TWIK-1* channels at depolarized potentials (Fig. 6a,b). These currents were sensitive to block by quinine, a known TWIK-1 inhibitor^7^,^9^, but were insensitive to block by 50-μM THexA (Fig. 6c, Supplementary Fig. 5).

We next measured currents for the L146N-mutant channel and unlike WT TWIK-1*, outward currents could be measured using K^+^. These currents were also enhanced by Rb^+^ (Fig. 6a). However, in marked contrast to WT TWIK-1*, the L146N channels were blocked >90% by 50-μM THexA and >70% by 1-μM THexA (Fig. 6c). Interestingly, we also observed that the Rb^+^ currents through the WT TWIK-1* channel activated at higher voltages compared with the L146N-mutant (Fig. 6b). This result is consistent with the greater electrical driving force required to overcome the energetic barrier created by a hydrophobic versus hydrophilic nanopore^33^. Overall, these results therefore strongly support the idea that the L146N mutation directly influences the conduction pathway deep within the inner cavity.

### Polar substitutions at Leu146 increase channel activity

**Figure 7:**
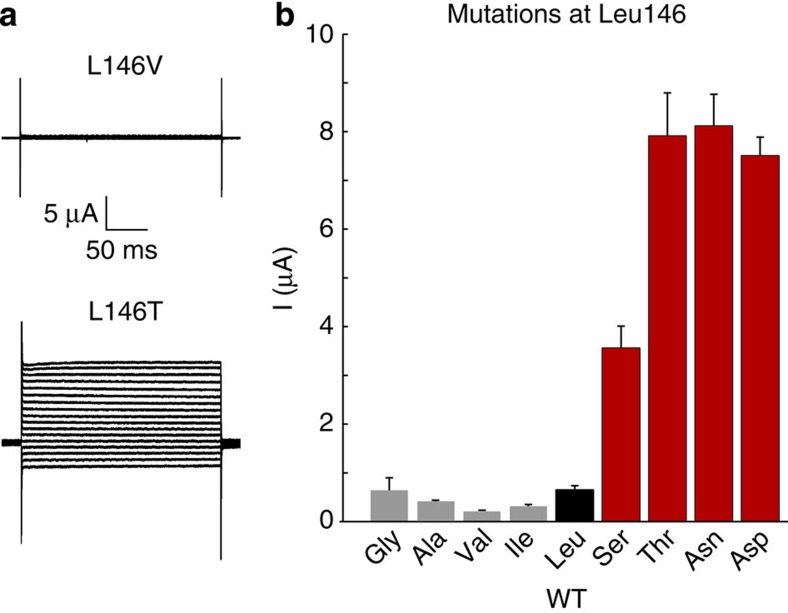
Representative current traces of TWIK-1* channels with different hydrophilic or hydrophobic substitutions at Leu146. (**a**) Currents were recorded from a series of voltage steps between −120 and +40 mV with a holding potential of −80 mV. (**b**) Mean currents at 0 mV for a range of different of hydrophobic (grey) and hydrophilic (red) substitutions at the Leu146 position (WT TWIK-1* is shown in black).

To further support this hypothesis we introduced a series of different hydrophilic and hydrophobic residues at Leu146 and examined their effect upon the functional properties of TWIK-1*. Only very small whole-cell currents could be recorded from WT TWIK-1* channels (Supplementary Fig. 4a). However, we found that when polar substitutions were made at Leu146, very large whole-cell currents could be recorded, but that hydrophobic side chains of varying sizes had no effect (Fig. 7). For example, the hydrophobic substitution L146V is similar to WT TWIK-1*, whereas the isosteric, but hydrophilic threonine substitution (L146T) produces large whole-cell currents equal in size to L146D or L146N. Likewise, while the L146A mutation was similar to wild-type TWIK-1*, large currents were observed with the isosteric, but hydrophilic, L146S mutation (Fig. 7). These results strongly support a direct correlation between the ability to hydrate this inner cavity and the functional activity of TWIK-1.

### Other polar substitutions within the hydrophobic cuff

**Figure 8:**
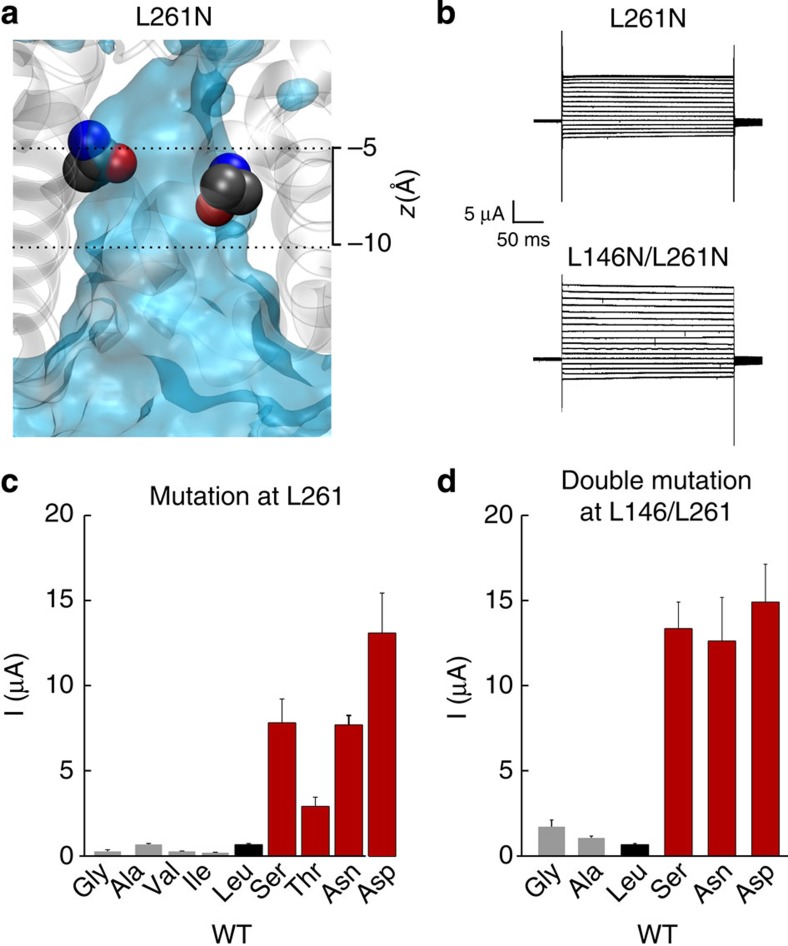
Hydrophilic substitutions at Leu261 also hydrate the inner pore. (**a**) Average water density of the L261N-mutant channel structure. The heavy atoms of the mutant side chains at the end of the MD simulation are shown as van der Waals spheres (see also Fig. 2c). Dashed lines represent approximate position of the hydrophobic cuff. (**b**) Representative whole-cell currents recorded from L261N and L146N-/L261N-mutant TWIK-1* channels. (**c**) Mean whole-cell currents recorded at 0 mV for a series of hydrophobic (grey) and hydrophilic (red) substitutions at the Leu261 position. WT TWIK-1* is indicated in black. (**d**) Combining mutations at both Leu146 and Leu261 markedly enhances whole-cell currents for hydrophilic, but not hydrophobic substitutions.

To further validate the role of this hydrophobic cuff, we used identical *in silico* and *in vitro* mutagenesis strategies to examine the other major side chain which contributes to the cuff, that is, Leu261 located on M4. Similar to the results observed for Leu146, we also found that isosteric polar substitutions at Leu261 (L261D and L261N) led to retention of water within the inner pore during MD simulations (Fig. 8a; Supplementary Fig. 2c,d). Importantly, these mutations also produced large whole-cell currents (Fig. 8b,c). Moreover, similar to the effect observed with Leu146, other small but polar substitutions at Leu261 also markedly increased whole-cell current levels, whereas isosteric non-polar mutations did not, for example, L261A versus L261S (Fig. 8c).

To examine the role of this hydrophobic barrier even further, we next introduced mutations at both Leu146 and Leu261, thereby allowing all four main side chains within the cuff to be changed. We found that the double polar substitutions (for example, L146S/L261S) produced large robust currents. However, a double non-polar alanine substitution (that is, L146A/L261A) did not increase currents, and even introduction of two glycines only led to a small enhancement of current levels (Fig. 8d).

Finally, we examined the effect of substitutions at Leu264, which also contributes to the hydrophobic cuff. The side chains of Leu264 are positioned at the interface between the M2 and M4 helices and so are not fully exposed to the conduction pathway; only one of the two δ-methyl groups of each side chain are exposed to the inner pore (Supplementary Fig. 6a). Nevertheless, we found that MD simulations of L264D mutant channels also led to retention of water within the inner pore (Supplementary Fig. 6b). Furthermore, hydrophilic substitutions at this position markedly increased whole-cell currents when compared with isosteric hydrophobic substitutions, consistent with a partial contribution of Leu264 to the hydrophobic nature of the inner pore.

## Discussion

Utilizing a combination of computational approaches supported by experimental validation, we now provide evidence that the hydrophobic nature of the TWIK-1 pore restricts full hydration of the inner cavity and that this generates an energetic barrier restricting ion permeation through these channels. These results are therefore consistent with the presence of a hydrophobic gate which is now an emerging concept in the field of ion channel biophysics^23^,^25^,^26^,^27^,^28^,^29^,^30^,^31^,^34^.

We validate this hypothesis by demonstrating, both computationally and functionally, that introduction of hydrophilic groups can directly disrupt this barrier. In particular, we demonstrate that the L146N mutation dramatically enhances the block of TWIK-1* currents by intracellular THexA. These quaternary ammonium ions have been shown to bind deep within the K2P channel pore in a region which would directly be affected by this hydrophobic barrier in TWIK-1 (ref. 18). The reduced voltage dependence of the L146N Rb^+^ currents is also consistent with recent experimental studies of the behaviour of ions and water in hydrophobic nanopores, where dewetting can be overcome in a voltage-dependent manner to induce current flow^33^,^35^. Also the relative hydrophobicity of the nanopores increased the voltage required to induce hydration^33^. This therefore supports our hypothesis that the L146N mutation directly alters the hydrophobicity of the conduction pathway.

The L146N mutation also does not appear to alter regulation of the channel by extracellular pH or to affect inhibition by quinine (Supplementary Fig. 5), and it has been shown that mutation of L146 does not alter the levels of TWIK-1* expression in oocytes^11^. Therefore, although we cannot exclude additional effects of these mutations on other modalities of TWIK-1 channel gating, we conclude that polar substitutions within this hydrophobic cuff increase TWIK-1 currents by directly disrupting this hydrophobic barrier to water and hydrated ions.

Unlike classical tetrameric K^+^ channels, the unusual dimeric architecture of the K2P channels places important structural constraints upon the ability of the pore-lining transmembrane helices to form a standard bundle-crossing gate. Evidence already exists that the selectivity filter plays a dynamic role in the control of TWIK-1 channel function^5^,^6^,^11^,^14^, but the role of additional structures in channel gating were unclear from the initial crystal structure of TWIK-1. Our demonstration that the C-helix remains stable, and that the cytoplasmic mouth of the pore remains open and hydrated in MD simulations, strongly suggests that the cytoplasmic mouth of the pore does not act as a gate as seen in Kv and Kir channels. Instead, our findings suggest that the hydrophobic inner pore of TWIK-1 acts as a strong barrier to ion permeation. In addition to the filter-gating mechanism, this may therefore contribute an additional mechanism to control the flow of ions through this channel.

It has been shown that in some cell types, post-translational sumoylation of Lys274 within the C-helix of TWIK-1 may account for its lack of functional activity^8^,^9^. However, when TWIK-1 is expressed in *Xenopus* oocytes, this effect still remains controversial^11^,^12^,^13^ (Supplementary Fig 4). This does not preclude a role for sumoylation of TWIK-1 in other cell types, but our results clearly demonstrate that the hydrophobic barrier we have identified in this study contributes to the low basal levels of functional activity associated with TWIK-1.

The physiological mechanisms which regulate TWIK-1 currents *in vivo* are still poorly understood and so it is not yet possible to address how modulation of this hydrophobic barrier might occur. However, by analogy to other K2P channels which also possess a large C-terminal cytoplasmic domain connected to the end of the M4 helix^36^, it is conceivable that regulatory inputs into this domain could induce subtle movements of the C-helix and pore-lining helices to modulate this hydrophobic barrier. It will also be interesting to examine whether similar hydrophobic barriers may exist in other K2P channels; these leucine residues are not highly conserved in other K2P channels, but there is an isoleucine residue at this position in M2 of THIK-2 and its mutation (I158D) markedly enhances channel activity^37^. Also, hydrophobic, but not polar, substitutions at the equivalent Leu146 position in M2 of the *Drosophila* KCNKØ channel cause reduced channel activity^38^.

Hydrophobic barriers and the concept of hydrophobic gating were first observed during MD simulations of model nanopores^23^,^24^,^25^,^26^,^27^. Subsequently, this process has been shown to play a critical role in the gating of several ligand-gated Cys-loop channels^31^,^34^, prokaryotic mechanosensitive channels^28^,^29^ and even voltage-gated K^+^ channels^30^. The biophysical basis of these hydrophobic gates has now been well-characterized; computational studies of Cys-loop channels show that the energy needed to hydrate a hydrophobic gate is greater than the energy needed to solvate an ion at this point, thereby allowing these regions to function as effective barriers to permeation^34^. Furthermore, in model nanopores hydration of the barrier can also be achieved by introduction of polar groups into the gate^27^. Similar results have been observed in both Cys-loop and MscS/MscL channels^28^,^29^,^31^,^34^.

The hydrophobic barrier we observe in TWIK-1 may also share some similarities with the deep pore gating mechanisms found in the SK, BK and MthK Ca^2+^-activated potassium channels^39^,^40^,^41^,^42^,^43^ which also appear to lack a bundle-crossing gate. In summary, we believe that these results not only provide an important insight into the mechanisms which control TWIK-1 K2P channel activity, but also provide further evidence of how the unusual behaviour of water in a confined space is an increasingly important consideration for our understanding of ion channel structure and function.

## Methods

### MD simulations

The TWIK-1 crystal structure (PDB ID: 3UKM, chains A and B) was used as the starting structure for the simulations. Missing atoms and loops (residues 94–99 between E2 and P1 and residue 169–174 between M2 and M3 (Supplementary Fig. 1) were modelled using Modeller9.9 software^34^,^44^. The resultant TWIK-1 models were converted to CG (Martini v2.1) representations and CGMD simulations, then run for 500 ns at 323 K to permit the assembly and equilibration of a bilayer containing 298 POPC lipids around the embedded membrane protein^45^,^46^. The protein and lipids were next converted to atomistic structures using the CG2AT method described previously^47^. The initial system was solvated with simple point charge (SPC) water and 150-mM NaCl. K^+^ ions were placed at positions S2 and S4 in the selectivity filter with an additional ion in the inner pore. Two water molecules were also added to the filter at the S1 and S3 positions. Initial atomistic simulations employed the GROMOS96 43a1 force field with SPC water at 323 K. The atomistic system was equilibrated for 1 ns with the non-hydrogen atoms of the protein restrained with a spring constant of 10 KJ mol^−1^ Å^−2^ at constant pressure (1 atm) and temperature, before the 100 ns unrestrained MD simulation with a timestep of 2 fs. Both the 1-ns positional restraint and 100-ns MD simulations were repeated by randomizing initial velocity to obtain two 100-ns simulation. *In silico* mutations were made using pyMOL mutagenesis scripts on the initial atomistic TWIK-1 system. The mutant systems were then energy minimized before the 1-ns positional restraint and 100-ns MD simulations.

To test the sensitivity of our MD findings, we repeated the atomistic simulations using two additional force fields, GROMOS 53A6 with SPC water, and optimized potentials for liquid simulations (OPLS) all-atom protein particles with united atom POPC lipids and TIP4P water. These simulations, along with our initial systems were also simulated at 310 K to also test temperature sensitivity. To determine whether dewetting precedes any alteration in inner pore structure, the Cα atoms were restrained with spring constant of 10 KJ mol^−1^ Å^−2^ and run for another 20 ns.

### MD analysis

Snapshots of MD simulations were sampled at every 0.1 ns and visualized using VMD, Chimera or Pymol. HOLE radius profiles were generated using MD analysis and HOLE^48^,^49^. Water occupancy at the hydrophobic constriction was analyzed by counting the number of water oxygen atoms at a region −5 Å<z<−10 Å below the S4 ion binding site (Thr117 and Thr225, which define the 0 Å position on the *z*-axis, see Fig. 1). Water occupancy data from two 100-ns simulations were then combined to calculate the mean (*μ*) *n*H2O values and to generate fractional occupancy histograms. Average water density maps were generated by using the Volmap plugin tool with three-dimensional grids every 0.5 Å for each simulation. The maps were then normalized to bulk water density and visualized at an isovalue of 0.5. Water occupancy of mutant channels were compared with WT by obtaining the difference in mean occupancy values Δ*μ* (Mutant–WT).

### Potential mean force calculations

Starting structures for one-dimensional potential mean force calculations were obtained from snapshots at 50 ns of unstrained equilibrium simulations in OPLS all-atom force fields. We picked starting structures which had similar radius profile between WT and L146N-mutant channel, but contained *n*H2O=0 for WT and *n*H2O=12 for L146N mutant (Fig. 5b). Twenty-five umbrella-sampling windows were produced by placing a K^+^ ion every 1 Å in the z direction from bulk water into the inner pore^24^. This K^+^ ion was harmonically restrained by a force constant of 10 KJ mol^−1^ Å^−2^ in the z plane and the system was simulated for 1.25 ns. The z position of this K^+^ ion, relative to centre-of-mass of the inner pore was sampled every 20 fs. WHAM v2.2 was used to calculate the probability mass function^50^.

### Molecular biology and electrophysiology

Wild-type and mutant TWIK-1 channel currents were studied by expression in *Xenopus* oocytes. The human TWIK-1 subunit was first cloned into the pBF oocyte expression vector, which adds the 5′ and 3′ untranslated regions of the *Xenopus* β-globin gene, and mutations were introduced by site-directed mutagenesis. The TWIK-1* channel has mutation of a dileucine trafficking motif (I293A/I294A), which permits stable expression in the plasma membrane, and was used as the background ‘wild-type’ template for all mutations^12^. Messenger RNA was transcribed *in vitro* and 15 ng of either wild-type or mutant subunits were injected. Oocytes were incubated for 12 h at 17 ^°^C in ND96 buffer at pH 7.4 (96 mM NaCl, 2 mM KCl, 2 mM MgCl_2_, 1.8 mM CaCl_2_, 5 mM HEPES), followed by a further 12 h in an adjusted ND96 buffer, where all sodium is replaced with potassium^11^. Two-electrode voltage clamp recordings were taken 24 h after injection using an OC-725C Oocyte Clamp amplifier (Warner Instruments), Digidata 1440 interface and pClamp10 software. For the recordings, the oocytes are perfused with the standard ND96 buffer at pH 7.4. A step protocol from −120 mV to +40 mV was used to record the currents for 300 ms, from a holding potential of −80 mV. Excised patch recordings were performed using an Axopatch 200B amplifier. The pipette solutions contained 116 mM KCl, 4 mM NaCl, 1.8 mM CaCl2 and 10 mM HEPES, and the bath solution contained 119 mM KCl, 1 mM NaCl, 2 mM EGTA and 10 mM HEPES. Both solutions were adjusted to pH 7.2. Where indicated, KCl was replaced by RbCl in the bath solution. Currents were recorded in an inside-out configuration, using a step protocol from −100 to +100 mV, with a holding potential of −80 mV.

## Author contributions

P.A. performed all computational studies. All other experimental work was performed by F.A-W. and G.B. The study was conceived by P.A. and supervised by M.S.P.S. and S.J.T. All authors were involved in the interpretation of the experimental studies. The manuscript was written by P.A. and S.J.T. with contributions from all other authors. P.A. and F.A-W. contributed equally to this study.

## Additional information

**How to cite this article:** Aryal, P. *et al.* A hydrophobic barrier deep within the inner pore of the TWIK-1 K2P potassium channel. *Nat. Commun.* 5:4377 doi: 10.1038/ncomms5377 (2014).

## Supplementary Material

Supplementary Information Supplementary Figures 1-6

Supplementary Movie 1 Dewetting of the TWIK-1 inner pore. Snapshots of water molecules (cyan) inside the inner pore of TWIK-1 channel taken every 0.1 ns during a 100 ns MD simulation. For orientation, the K+ ion at the bottom of the selectivity filter (S4) is shown as a purple sphere for orientation. Water loss is seen deep within the inner pore, but not at the cytoplasmic mouth of the channel.
