# Supplementary material for: A hydrophobic barrier deep within the inner pore of the TWIK-1 K2P potassium channel
Source: Nat Commun. 2014 Jul 8;5:4377. doi: 10.1038/ncomms5377 (PMC4102122; doi:10.1038/ncomms5377)
Supplement: Supplementary Information — Supplementary Figures 1-6 [file ncomms5377-s1.pdf]

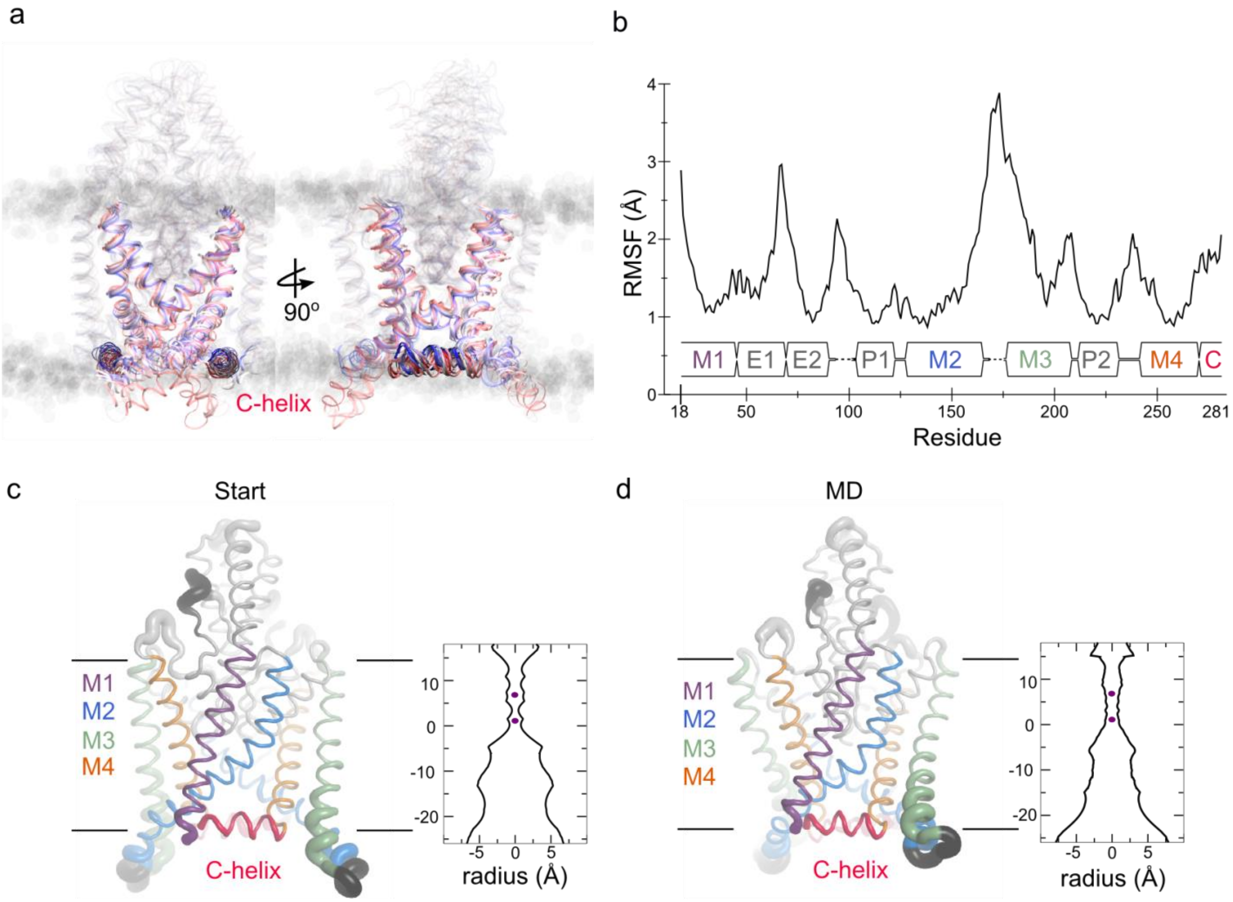

Supplementary Figure 1

TWIK-1 stability during MD simulations in a phospholipid bilayer. (a) Snapshots every 10 ns from a 100 ns MD simulation colored red (start) to blue (end) showing the positions of M2 M4 and the c-helix. Snapshots show the C-helix remains stable and parallel to the membrane. **(b)** Average root mean square fluctuations (RMSF) calculated for backbone atoms of both chains from two 100ns simulations with a schematic of the domain architecture of TWIK-1. Dashed lines represent missing regions of the original 3UKM crystal structure. **(c) left:** Schematic worm representation of the starting structure scaled to depict the range of fluctuations during two 100ns simulations with the TM domains. Largest fluctuations are away from the pore and near regions missing from the crystal structure (shown in black) **right:** radius profile of the ion conduction pathway at the start of MD simulation. The zero position on the z-axis is defined by the position of Thr117 and Thr225 which form the S4 binding site **(d) Left:** Worm representation of the average coordinates of the protein from 2x 100 ns MD simulations. **Right:** average radius during the MD simulation.

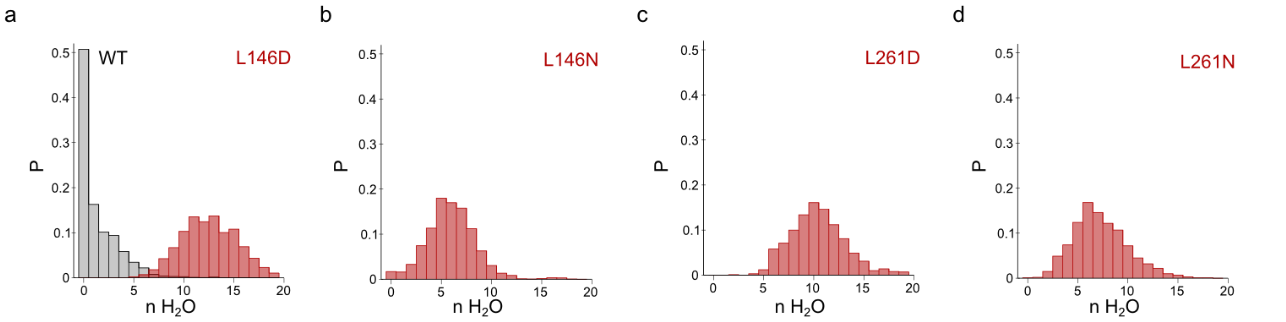

Supplementary Figure 2

Isosteric hydrophilic substitutions in the hydrophobic cuff of TWIK-1 increase water occupancy. Normalized histogram of water molecules present within the hydrophobic cuff region for two 100ns MD simulations sampled every 0.1 ns for **(a)** L146D (μ nH_2O = 13.2) overlaid on those for the WT simulation (grey, taken from Fig. 2c), **(b)** L146N (μ nH_2O = 6.97), **(c)** L261D (μ nH_2O =11.41), and **(d)** L261N (μ nH_2O = 9.44). For each mutant there is a large increase in the number of waters present and this region remains hydrated throughout the simulation. This contrasts to the WT channel where the average nH_2O is 1.95, and water is absent >50% of the time.

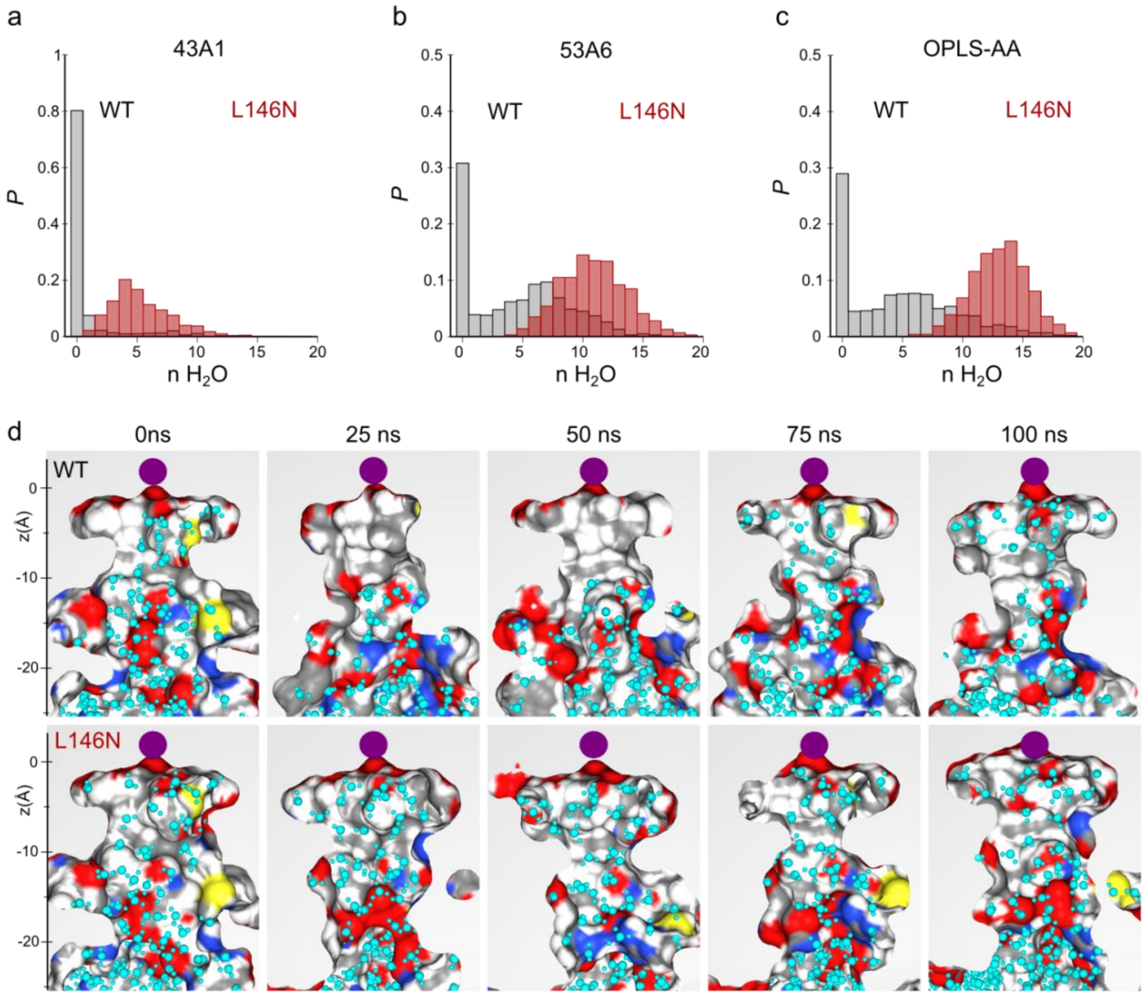

Supplementary Figure 3

Dewetting of the hydrophobic inner pore in different force fields. The principal findings of our simulations are reproducible in several different force fields. Normalized histograms of water within the hydrophobic cuff are shown for 100 ns MD simulations of WT and L146N mutants at 310K in **(a)** Gromos 43A1 with SPC water ($\Delta\mu$ nH_2O $_{(L146N\text{-}WT)}$ = 5.25) **(b)** 53A6 with SPC water ($\Delta\mu$ nH_2O $_{(L146N\text{-}WT)}$ = 6.5, and **(c)** the OPLS all atom force field with TIP4P water ($\Delta\mu$ nH_2O $_{(L146N\text{-}WT)}$ = 8.4. For each force field there is a large increase in the number of waters present for L146N mutant vs WT ($\Delta\mu$ nH_2O >5), and L146N mutant channels remains hydrated through the simulations. (**d**) Snapshots of sections through the inner pore during MD simulation using the OPLS all atom force field with TIP4P water for both WT TWIK-1 and L146N mutant structures. Dewetting of the inner pore is visible in the WT TWIK-1, but not the mutant L146N channel. Quantification of this effect is shown in panel (c). Note that in both structures the cytoplasmic mouth of the inner pore is hydrophilic and remains hydrated throughout the simulations.

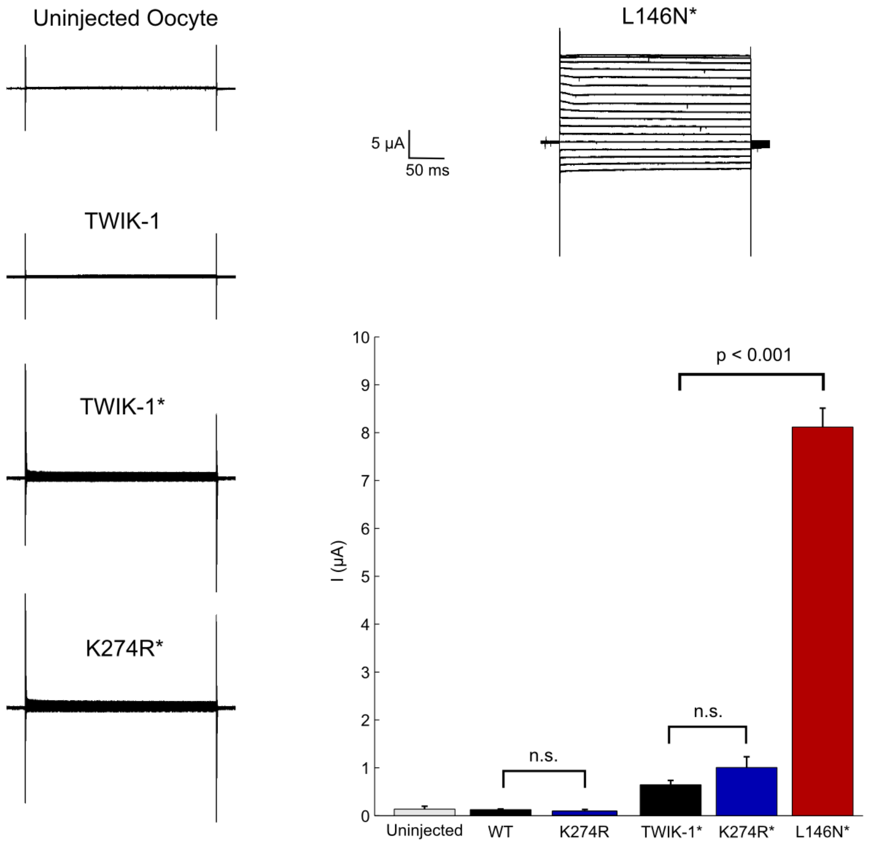

Supplementary Figure 4.

Mutation of Leu146 increases whole-cell currents. Representative whole-cell currents are shown for an uninjected oocyte and from oocytes expressing wild-type TWIK-1 or the trafficking mutant TWIK-1* (i.e. I293A/I294A) which prevents rapid endocytosis from the membrane (refs 10, 11). Currents recorded from wild-type TWIK-1 are not significantly different from uninjected oocytes. Very small currents (<0.5 µA) can be recorded from the wild-type TWIK-1* channels whereas the L146N TWIK-1* mutant channels (L146N*) express large whole-cell currents. When expressed in oocytes, the K274R mutation in WT TWIK-1 (which prevents sumoylation of Lys274) does not produce any increase in currents, nor does it produce any significant increase in the TWIK-1* mutant channel which is stable within the membrane (K274R*), therefore factors other than sumoylation must be responsible for the low levels of TWIK-1* basal currents in oocytes. All whole cell currents shown were recorded in ND 96 bath solution using voltage steps from -120 mV to +40 mV from a holding potential of -80 mV. Averaged currents values were measured at 0 mV and significance measured using a paired students t-test.

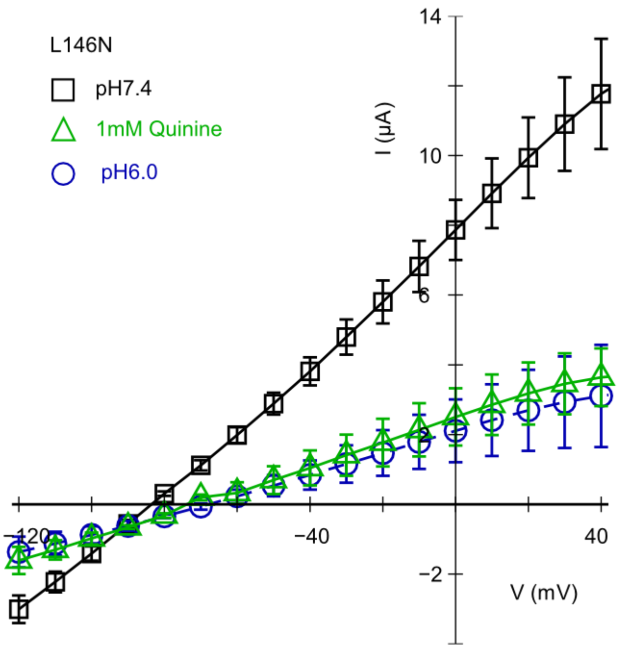

Supplementary Figure 5.

Mutation of Leu146 does not affect inhibition by quinine or external pH. Whole-cell currents recorded from L146N TWIK-1* mutant channels. Wild-type TWIK-1 can he inhibited by external acidification and quinine (refs. 5, 7, 9, 10). Here we show that the L146N mutant channels still exhibit inhibition by external acidification as well as application of 1 mM quinine. This demonstrates that mutations within the hydrophobic cuff do not affect the external pH-gating machinery which is thought to reside close to or within the selectivity filter.

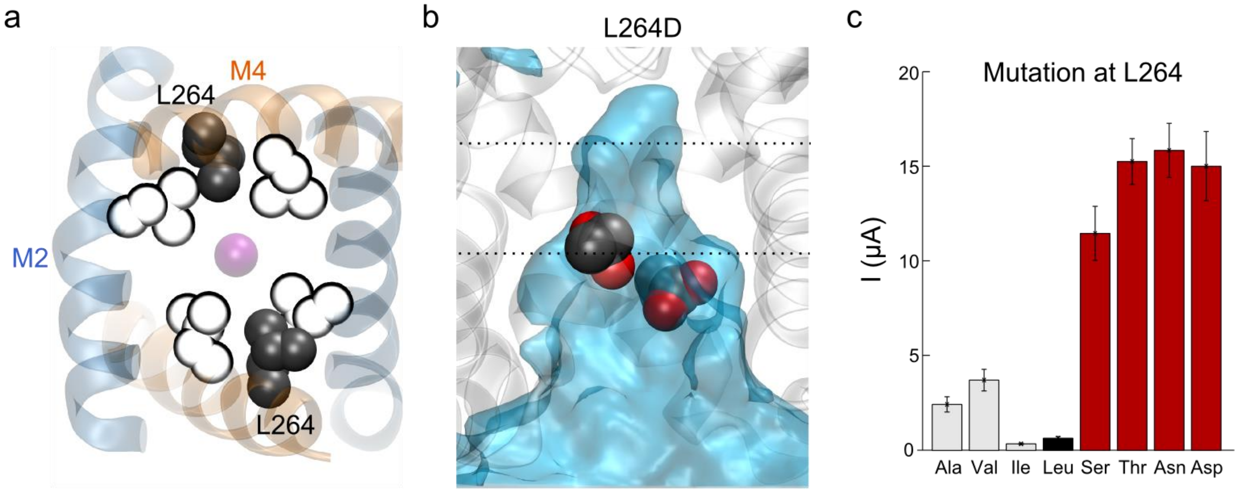

Supplementary Figure 6.

Leu264 also contributes to the hydrophobic cuff. (**a**) bottom-up view of the hydrophobic cuff with Leu264 shown in grey. Leu146 and Leu261 are shown (in white) as in Fig. 3b. Leu264 (in grey) is at the interface between M2 and M4 and only one δ-methyl group of the Leu264 side chain is exposed to the inner pore. (**b**) Average water density of the L264D mutant simulation represented as in Fig. 4a. (**c**) Mean currents at 0 mV of series of hydrophobic (grey) and hydrophilic (red) substitutions at Leu264 in TWIK-1*.
